# Evolving a New Efficient Mode of Fructose Utilization for Improved Bioproduction in *Corynebacterium glutamicum*

**DOI:** 10.3389/fbioe.2021.669093

**Published:** 2021-05-28

**Authors:** Irene Krahn, Daniel Bonder, Lucía Torregrosa-Barragán, Dominik Stoppel, Jens P. Krause, Natalie Rosenfeldt, Tobias M. Meiswinkel, Gerd M. Seibold, Volker F. Wendisch, Steffen N. Lindner

**Affiliations:** ^1^Chair of Genetics of Prokaryotes, Faculty of Biology and CeBiTec, Bielefeld University, Bielefeld, Germany; ^2^Systems and Synthetic Metabolism, Max Planck Institute of Molecular Plant Physiology, Potsdam-Golm, Germany; ^3^Institute of Biochemistry, University of Cologne, Cologne, Germany; ^4^Department of Biotechnology and Biomedicine, Technical University of Denmark, Lyngby, Denmark

**Keywords:** metabolic engineering, synthetic biology, PTS, NADPH, lysine, fructose, adaptive laboratory evolution

## Abstract

Fructose utilization in *Corynebacterium glutamicum* starts with its uptake and concomitant phosphorylation *via* the phosphotransferase system (PTS) to yield intracellular fructose 1-phosphate, which enters glycolysis upon ATP-dependent phosphorylation to fructose 1,6-bisphosphate by 1-phosphofructokinase. This is known to result in a significantly reduced oxidative pentose phosphate pathway (oxPPP) flux on fructose (∼10%) compared to glucose (∼60%). Consequently, the biosynthesis of NADPH demanding products, e.g., L-lysine, by *C. glutamicum* is largely decreased when fructose is the only carbon source. Previous works reported that fructose is partially utilized *via* the glucose-specific PTS presumably generating fructose 6-phosphate. This closer proximity to the entry point of the oxPPP might increase oxPPP flux and, consequently, NADPH availability. Here, we generated deletion strains lacking either the fructose-specific PTS or 1-phosphofructokinase activity. We used these strains in short-term evolution experiments on fructose minimal medium and isolated mutant strains, which regained the ability of fast growth on fructose as a sole carbon source. In these fructose mutants, the deletion of the glucose-specific PTS as well as the 6-phosphofructokinase gene, abolished growth, unequivocally showing fructose phosphorylation *via* glucose-specific PTS to fructose 6-phosphate. Gene sequencing revealed three independent amino acid substitutions in PtsG (M260V, M260T, and P318S). These three PtsG variants mediated faster fructose uptake and utilization compared to native PtsG. In-depth analysis of the effects of fructose utilization *via* these PtsG variants revealed significantly increased ODs, reduced side-product accumulation, and increased L-lysine production by 50%.

## Introduction

Canonical metabolic routes evolved for superior performance in the natural habitat but often they do not represent the ideal choice from a biotechnological perspective (Erb et al., 2017). If more suitable alternative pathways are known, rational approaches of metabolic engineering can redirect metabolic pathways into more advantageous directions. In the absence of a known and better-suited natural alternative, adapted laboratory evolution (ALE) may select for efficient pathway variants. *Corynebacterium glutamicum* is employed in the million-ton scale bioproduction of amino acids, with the lion’s share split between L-glutamate and L-lysine (Wendisch, 2020). Beyond amino acids, amines, organic acids, and alcohols are produced with this bacterium (Becker et al., 2018; Mindt et al., 2020).

NADPH is an important cofactor for anabolic reactions and hence a limiting factor in the production of metabolites with a particularly high demand for NADPH, e.g., L-lysine, which requires four molecules of NADPH per molecule of L-lysine produced (Marx et al., 1996). To provide NADPH, *C. glutamicum* possesses several dehydrogenases, which use NADP^+^ as cofactor. These are the glucose 6-phosphate dehydrogenase (Zwf), and the 6-phosphogluconate dehydrogenase (Gnd) of the oxidative part of the pentose phosphate pathway (oxPPP), the isocitrate dehydrogenase (Icd) in the TCA cycle, and the malic enzyme (MalE), and their overexpression improved production of L-lysine (Georgi et al., 2005; Becker et al., 2007). NADPH provision was optimized by heterologous expression of genes encoding the membrane-bound transhydrogenase from *Escherichia coli* (Kabus et al., 2007). Although *C. glutamicum* lacks transhydrogenase, it is known to run an ATP-consuming transhydrogenase-like cycle between the anaplerotic reactions, malate dehydrogenase, and MalE, transferring electrons from NADH to NADP^+^ (Blombach et al., 2011). However, the predominant way of NADPH generation is *via* the oxPPP.

Metabolic engineering was used to broaden the substrate spectrum of *C. glutamicum* toward second generation feedstocks such as non-food wastes from biodiesel (glycerol) (Rittmann et al., 2008) or hemicellulose biomasses (xylose and arabinose) (Zhao et al., 2018). Yet, the sugars glucose, derived from starch hydrolysates as well as sucrose and fructose derived from molasses, are still the preferred carbon sources for amino acid production (Anaya-Reza and Lopez-Arenas, 2017). The decrease in L-lysine titers when fructose is used instead of glucose is drastic, resulting in a 40–75% lower L-lysine yield (Georgi et al., 2005). Although the entry points of glucose and fructose are only two reactions apart, the fluxes through the oxPPP, and hence the prevalent NADPH generating reactions, are significantly different. On fructose, a very low flux is described (10%), whereas glucose leads to a high flux (60%) (Kiefer et al., 2004). As a result, a low L-lysine product yield is reached on fructose compared to glucose (Georgi et al., 2005). Moreover, the reported high oxPPP fluxes on glucose may still be limiting for overproduction of high NADPH-consuming products, such as amino acids (Murai et al., 2020).

**FIGURE 1:**
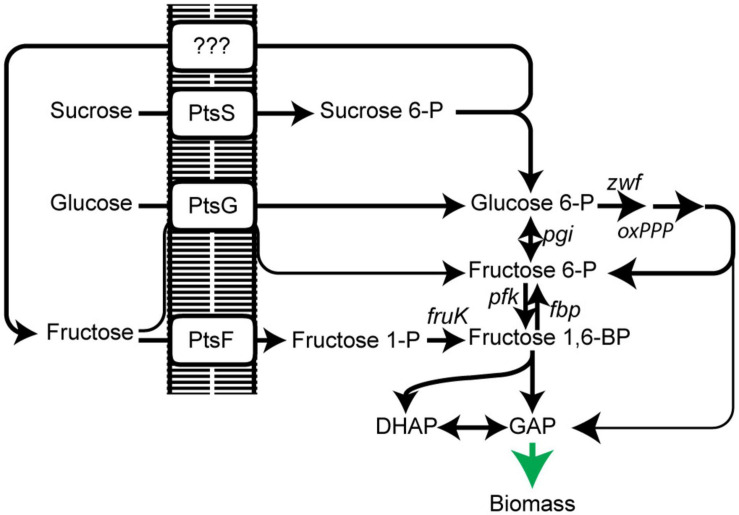
Scheme of PTS-dependent uptake and utilization of sugars sucrose, glucose, and fructose in *C. glutamicum*. PtsS, sucrose PTS; PtsG, glucose PTS; PtsF, fructose PTS; oxPPP, oxidative pentose phosphate pathway; pgi, phosphoglucose isomerase; pfk, 6-phosphofructokinase; fbp, fructose 1,6-bisphosphatase; fruK, 1-phosphofruktokinase; DHAP, dihydroxyacetone phosphate; GAP, glyceraldehyde 3-phosphate. Data represent one of two individual cultivations, which differed <5%.

While for glucose an ATP-dependent pathway, which can replace the phosphoenolpyruvate-dependent phosphotransferase system (PTS), is present in *C. glutamicum* (Moon et al., 2007; Lindner et al., 2011), fructose and sucrose are exclusively phosphorylated by the PTS (Ikeda, 2012). Glucose is phosphorylated to glucose 6-P by a glucose-specific PTS compound (PtsG). Sucrose is phosphorylated to sucrose 6-P *via* its PTS (PtsS) and subsequently cleaved to glucose 6-P and fructose; the latter is exported outside *via* a so far unidentified exporter. Fructose, regardless if added to the medium as a carbon source or originating from sucrose catabolism, is taken up and phosphorylated to fructose 1-P by a fructose specific PTS (PtsF) (Dominguez and Lindley, 1996; Parche et al., 2001). After a second ATP-dependent phosphorylation of fructose 1-P catalyzed by 1-phosphofructokinase (FruK), it enters glycolysis at the level of fructose 1,6-BP. Additionally, a minor fraction of fructose (<10%) is taken up and phosphorylated by PtsG to generate fructose 6-phosphate (Kiefer et al., 2004; Figure 1). Most strikingly, overexpression of fructose 1,6-bisphosphatase increased L-lysine production when fructose was used as the carbon source (Georgi et al., 2005), pointing to an advantage of fructose 6-P over fructose 1,6-BP for increasing oxPPP-flux as fructose 6-P is rapidly converted to glucose 6-phosphate by phosphoglucoisomerase and, consequently, increasing NADPH regeneration and productivity. Thus, shifting the carbon flux slightly closer to the entry point of the oxPPP allows a higher flux through the oxPPP and a higher NADPH regeneration rate. Similarly, L-lysine production from molasses was optimized by overexpression of fructose 1,6-bisphosphatase and fructokinase (Xu et al., 2013).

Here, we aimed at increasing the efficiency of the PtsG-catalyzed conversion of fructose to fructose 6-P. We generated strains unable to utilize fructose *via* its usual route and selected fast-growing strains after short-term evolution in fructose minimal medium. Isolated PtsG variants were identified and reverse engineering complemented fructose utilization in the deletion strains. Deletion of 6-phosphofructokinase in the mutants and overexpression of the PtsG variants in a fructose 1,6-bisphosphatase deletion strain confirmed fructose phosphorylation to fructose 6-P by the PtsG variants. ^13^C-labeling experiments revealed that a higher oxPPP flux is present in the reverse-engineered strains. Finally, the alternative way of fructose utilization was tested on L-lysine production, showing an increase in L-lysine yield from fructose.

## Materials and Methods

### Strains and Plasmids Used

*Corynebacterium glutamicum* strains and plasmids used are listed in Tables 1, 2, respectively. For plasmid construction, the primers listed in Supplementary Table 1 were used. For cloning, genes were amplified from genomic DNA and cloned by the indicated restriction sides (Supplementary Table 1) into similarly restricted pVWEx1. Deletion plasmids were constructed by cloning PCR-fused products of primer pairs A + B and C + D and cloned blunt-ended into *Sma*I-digested pK19mobsacB.

**TABLE 1:** *Corynebacterium glutamicum* strains used in this study.

**Strain**	**Deletion of gene, function**	**Derived from**	**Source**
*C. glutamicum*	Wild-type (WT), ATCC13032	–	ATCC
Δ*ptsF*	*ptsF*, fructose specific PTS compound	WT	Radek et al., 2016
Δ*ptsF*Δ*ptsG*	*ptsG*, glucose-specific PTS compound	Δ*ptsF*	This study
Δ*fruK1*	*fruK1*, 1-phosphofructokinase 1	WT	This study
Δ*fruK2*	*fruK2*, 1-phosphofructokinase 2	WT	This study
Δ*fruK1*Δ*fruK2*	*fruK1*, *fruK2*, 1-phosphofructokinase 1 + 2	Δ*fruK1*	This study
Δ*fbp*	*fbp*, fructose 1,6-bisphosphatase	WT	Rittmann et al., 2003
Δf*ruK1*Δ*fruk2*Δ*pfk*	*pfk*, 6-phosphofructokinase	Δ*fruK1*Δ*fruK2*	This study
Δ*fruK1*Δ*fruk2*Δ*ptsG*	*ptsG*, glucose-specific PTS compound	Δ*fruK1*Δ*fruK2*	This study
Δ*fruK1*Δ*fruk2*Δ*hpr*	*hpr*, general PTS compound Hpr	Δ*fruK1*Δ*fruK2*	This study
CgLYS4	DM1729 Δ*pta-*ac*kA* Δ*cat* Δace*AB* Δ*ldhA* Δ*nanR*	DM1729	Sgobba et al., 2018
CgLYS4 Δ*ptsF*	CgLYS4 deleted in Δ*ptsF*	CgLYS4	Sgobba et al., 2018

**TABLE 2:** Plasmids used in this study.

**Name**	**Properties/use**	**Source**
pVWEx1	Km^*R*^, pHM1519, *P*_*tac*_, *lacI*^*q*^, for IPTG inducible expression	Peters-Wendisch et al., 2001
pVWEx1-*ptsG*	pVWEx1, carrying WT *ptsG*	This study
pVWEx1-*ptsG^*M*260*V*^*	pVWEx1, carrying *ptsG* mutated in M260V	This study
pVWEx1-*ptsG^*M*260*T*^*	pVWEx1, carrying *ptsG* mutated in M260T	This study
pVWEx1-*ptsG^*P*318*S*^*	pVWEx1, carrying *ptsG* mutated in P318S	This study
pVWEx1-*ptsF*	pVWEx1, carrying WT *ptsF*	This study
pVWEx1-*lysC*^*fbr*^	pVWEx1, carrying feedback resistant version of *lysC* (T311I)	This study
pK19*mobsacB*	Km^*R*^, RP4; *mob*; oriV_*Ec*_; *sacB; lacZ*α*;* for allelic exchange	Schafer et al., 1994
pK19*mobsacB*Δ*fruK1*	pK1*9mobsacB* based for *fruK1* deletion	This study
pK19*mobsacB*Δ*fruK2*	pK1*9mobsacB* based for *fruK2* deletion	This study
pK19*mobsacB*Δ*pfk*	pK1*9mobsacB* based for *pfk* deletion	This study
pK19*mobsacB*Δ*ptsG*	pK1*9mobsacB* based for *ptsG* deletion	This study
pK19*mobsacB*Δ*ptsF*	pK1*9mobsacB* based for *ptsF* deletion	Radek et al., 2016
pK19*mobsacB*Δ*hpr*	pK1*9mobsacB* based for *hpr* deletion	Lindner et al., 2011

### Culture Conditions and Growth Experiments

*Corynebacterium glutamicum* strains were cultivated in LB (1% NaCl, 1% tryptone, and 0.5% yeast extract) or CgXII minimal medium [20 g/L (NH_4_)_2_ SO_4_, 5 g/L urea, 1 g/L KH_2_PO_4_, 1 g/L K_2_HPO_4_, 42 g/L MOPS, 10 mg/L CaCl_2_, 250 mg/L MgSO_4_ × 7 H_2_O, 0.01 mg/L FeSO_4_ × 7 H_2_O, 0.01 mg/L MnSO_4_ × 7 H_2_O, 0.001 mg/L ZnSO_4_ × 7 H_2_O, 0.0002 mg/L CuSO_4_, and 0.00002 mg/L NiCl_2_ × 6 H_2_0, pH 7) (Eggeling and Bott, 2005). For growth experiments, the strains grew in 50-ml LB cultures overnight, harvested by centrifugation (3220 × *g*), washed twice in CgXII without carbon source, and inoculated to an optical density (OD) of 1 in 50 ml of CgXII containing the indicated carbon sources. For plasmid construction, *E. coli* DH5α was used and cultured in LB medium. Precultures for growth experiments with *C. glutamicum* and all *E. coli* cultures were carried out in LB. For selection on pVWEx1 and derivatives, 50 and 25 mg/ml kanamycin was added to *E. coli* and *C. glutamicum* cultures, respectively. CgXII minimal medium (Eggeling and Bott, 2005) was used for growth, sugar uptake, and L-lysine production experiments. Cells were harvested in the exponential growth phase by centrifugation (RT, 3220 × *g* for 10 min) and washed twice in CgXII medium without carbon source. Gene expression was induced by addition of up to 1 mM isopropyl β-D-1-thiogalactopyranoside (IPTG). Ideal concentration of IPTG for *ptsF*/*G* expression was determined to be at 30 μM IPTG. Cultivations were carried out in 50-ml solutions in 500-ml baffled shaking flasks at 120 rpm and 30°C.

### Analysis of Sugars and Organic Acid Concentration, and Amino Acid Production

Lysine production: to verify L-lysine production, strains were inoculated to OD_600_ of 1 in CgXII media supplXemented with 4% fructose (w/v), 30 μM IPTG, and, if carrying a pVWEx1 variant, 25 μg/ml kanamycin in 500-ml baffled shake flasks. Supernatants were collected at 4, 8, 12, 24, 48, and 72 h after inoculation. L-lysine concentrations were determined in up to 1:5000 serial dilution of supernatants using an ICS-6000 HPIC Ion Chromatography equipped with an AminoPac PA10 IC column, ICS-6000 CD Conductivity Detector, and ADRS 600 Anion Dynamically Regenerated Suppressor (Dionex, CA, United States). The column was set with a 10–250 mM NaOH gradient at a 0.25 ml/min flow rate. Sugars and organic acid concentrations were quantified *via* HPLC as described previously (Rittmann et al., 2008).

### ^13^C Isotopic Labeling of Proteinogenic Amino Acids

^13^C-isotope tracing was performed to indirectly analyze carbon flux. Cells were cultured in 4 ml of CgXII medium containing ^13^C-1-glucose or ^13^C-1-fructose (Sigma-Aldrich, Taufkirchen, Germany) as sole carbon sources. Cultures were inoculated from CgXII + 20 mM pyruvate overnight cultures to an OD_600_ 0.01 and grown at 30°C until early stationary phase. Before inoculation, cells were washed twice (RT, 6000 × *g*, 3 min) in carbon source free CgXII medium. 10^9^ cells (∼1 ml of OD_600_ = 1) were pelleted, washed with ddH_2_O, and hydrolyzed in 1 ml of 6N hydrochloric acid at 95°C for 24 h. Subsequently to hydrolysis, HCl was evaporated by heating at 95°C under an air stream. Hydrolyzed biomass was resuspended in 1 ml of ddH_2_O. Amino acid masses were analyzed after separation by ultra-performance liquid chromatography (Acquity, Waters, Milford, MA, United States) using a C18-reversed-phase column (Waters, Eschborn, Germany) as previously described (Giavalisco et al., 2011). Mass spectra were acquired by an Exactive mass spectrometer (Thermo Scientific, Dreieich, Germany). Data were analyzed using Xcalibur (Thermo Scientific, Dreieich, Germany). Amino acid standards (Merck, Darmstadt, Germany) were used to determine specific retention times.

### Sugar Uptake Measurements

For ^14^C-labeled fructose uptake studies, strains were grown to early exponential growth phase with 50 mM fructose as sole carbon source and 30 μM IPTG, if appropriate. Cells were harvested by centrifugation, washed two times in ice-cold CgXII medium (without carbon source), resuspended to an optical density OD_600_ of 2 in CgXII medium, and stored on ice until measurement. Prior to the transport assay, cells were incubated for 3 min at 30°C. The assay was started by addition of 1 μM to 1 mM ^14^C-labeled fructose (specific activity of 45 mCi mmol^–1^; Hartmann Analytik, Braunschweig, Germany). At given time intervals (15, 30, 45, 60, and 120 s), 200-μl samples were filtered through glass fiber filters (type F; Millipore, Eschborn, Germany) and washed twice with 2.5 ml of 100 mM LiCl. The radioactivity of the filter samples was determined using scintillation fluid (Rotiszinth; Roth, Germany) and a scintillation counter (LS 6500; Beckmann, Krefeld, Germany).

## Results

### Construction and Characterization of Strains Lacking Fructose-Specific PTS and 1-Phosphofructokinase Genes

**FIGURE 2:**
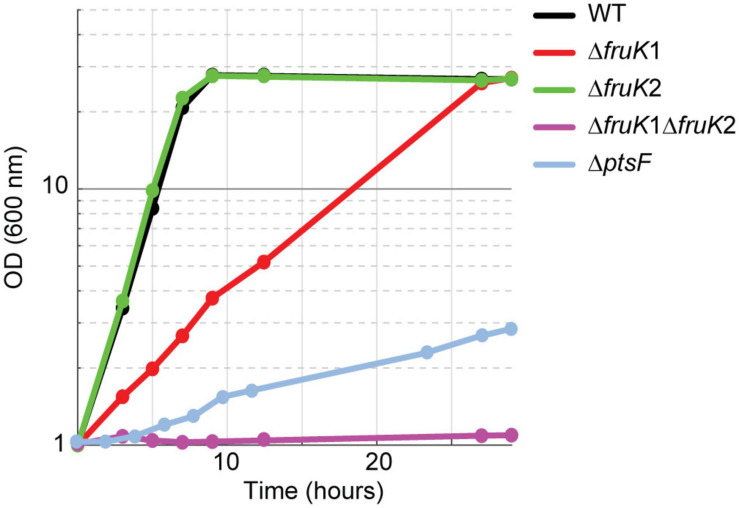
Growth on 2% fructose of strains lacking parts of the canonical fructose utilization pathway, fructose-specific PTS (*ptsF*), or 1-phosphofructokinases (*fruK1*, *fruK2*). Data represent one of two individual cultivations that differed <5%.

Previous studies suggested a PtsG-mediated fructose utilization to fructose 6-P in *C. glutamicum* (Kiefer et al., 2004). We expected that the direct generation of fructose 6-P instead of fructose 1,6-BP from fructose increases oxidative PPP flux leading to higher NADPH availability, which is advantageous for high NADPH-demanding bioproductions, such as L-lysine. In fact, overexpression of fructose 1,6-bisphosphatase increased L-lysine production from fructose (Georgi et al., 2005). The aim of this study was to explore the promiscuous reaction of PtsG and evolve it for increased activity. To be able to select for this route of fructose utilization and to evolve it, we generated two strains, which are deficient in the canonical route of fructose utilization. The first lacks the fructose-specific PTS compound (Δ*ptsF*), and the second lacks 1-phosphofructokinase activity (Δ*fruK1* Δ*fruK2*) (Figure 1). As expected, growth of these strains on fructose was strongly affected. The Δ*ptsF* strain grew with a very low growth rate and the Δ*fruK1* Δ*fruK2* strain did not grow at all within 24 h. Growth analysis of the individual 1-phosphofructokinase deletion strains on fructose revealed no effect by the deletion of *fruK2*, but slower growth when *fruK1* was deleted (Figure 2). Similarly to effects observed for Δ*ptsF* and Δ*fruK1* Δ*fruK2*, an *E. coli* 1-phosphofruktokinase deletion strain was reported to be unable to grow on fructose, and moreover growth on other carbon sources of this strain was inhibited when fructose or fructose 1-P were added to the medium, indicating a growth perturbing effect of fructose 1-P caused by regulatory or inhibitory function of this metabolite (Ferenci and Kornberg, 1973). In *C. glutamicum*, fructose 1-P acts as a negative effector of sugar regulator SugR and hence accumulation in the absence of 1-phosphofructokinase activity might affect sugar uptake and utilization (Dietrich et al., 2009), thus causing the growth difference of Δ*ptsF* and Δ*fruK1* Δ*fruK2* observed here. Both, Δ*ptsF* and Δ*fruK1* Δ*fruK2* were considered suitable for performing shake-flask short-term evolution experiments. In particular, the slow growth of Δ*ptsF* indicates the presence of an alternative way for fructose utilization in our background strain, suggesting a good starting point for optimization of the reaction through evolution. In contrast to the results obtained with Δ*ptsF*, a deletion strain lacking the general PTS compound HPR is unable to grow on fructose, also after prolonged incubation, pointing to the contribution of PtsG as reported earlier (Kiefer et al., 2004; Moon et al., 2007) (data not shown).

### Adaptive Evolution for Growth on Fructose

To evolve the specificity of the glucose-specific PTS compound toward fructose, *C. glutamicum* strains Δ*ptsF* and Δ*fruK1*Δ*fruK2* were incubated in CgXII minimal media containing 2% fructose (w/v) as a sole carbon source. After incubation for 3–4 days, all strains had grown to stationary phase. Samples from each culture were transferred to LB plates for single colony isolation. When subsequently transferred to fructose minimal medium, the isolated strains immediately showed fast growth, indicating that a mutation compensating for the growth deficiency had occurred. To increase the variance, 20 cultures of each genetic background (Δ*ptsF* and Δ*fruK1*Δ*fruK2*) were incubated for 4 days in fructose minimal medium. All strains reached stationary phase within this time. To identify if mutations in PtsG are responsible for the growth recovery, the *ptsG* locus of the isolated mutants was amplified by PCR and sequenced by Sanger sequencing. Sequencing results revealed that all strains analyzed (*n* = 40) had non-synonymous substitution in the coding sequence of *ptsG*. Among these mutants, only three different point mutations were found. These mutations altered the amino acids M260V, M260T, or P318S. The most abundant mutation among the three was M260V (Supplementary Figure 1).

**FIGURE 3:**
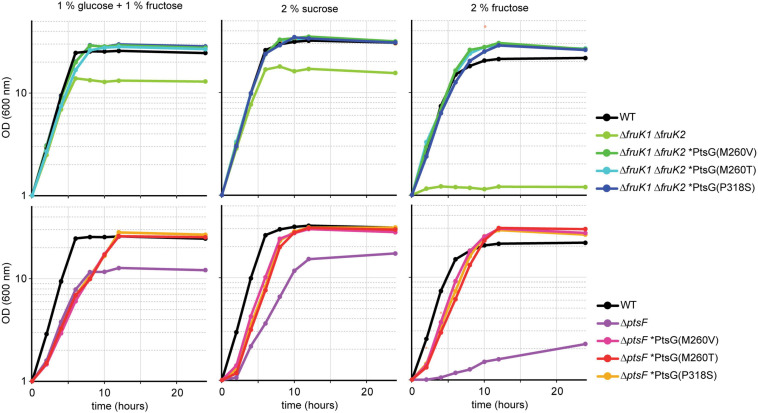
Growth of isolated fructose mutants on glucose + fructose, sucrose, and fructose. Data represent one of two individual cultivations that differed <5%.

Mutants from both the Δ*ptsF* and the Δ*fruK1* Δ*fruK2* background representing all three PtsG variants were analyzed for growth in fructose, sucrose, as well as in fructose + glucose minimal medium (Figure 3). The six analyzed mutants showed restored, fast growth with fructose as a sole source of carbon; moreover, they grew to slightly higher ODs than the WT strain. In sucrose minimal medium as well as in glucose + fructose medium, the strains grew similarly to the WT control and reached ODs twofold higher than their parental strains (Δ*ptsF* or Δ*fruK1*Δ*fruK2*), since the latter can only efficiently utilize the glucose part of the provided carbon sources. While the mutant strains reached comparable maximal ODs in medium containing sucrose only, they grew to slightly higher maximal ODs with glucose + fructose and to significantly higher maximal ODs when fructose was used as the sole carbon source.

After having shown that the mutants derived from the parental Δ*fruK1* Δ*fruK2* strain grew with fructose and contained non-synonymous mutations in the *ptsG* locus, either the gene encoding the general PTS subunit *hpr* or the glucose-specific subunit PtsG was deleted in these mutants. Both deletions *ptsG* and *hpr* in the Δ*fruK1* Δ*fruK2* mutants abolished growth with fructose in these strains (Supplementary Figure 2). Thus, the activity of the glucose-specific PTS is responsible for fructose utilization in these mutants.

### Evidence for Generation of Fructose 6-Phosphate From Fructose *via* Glucose-Specific PTS

**FIGURE 4:**
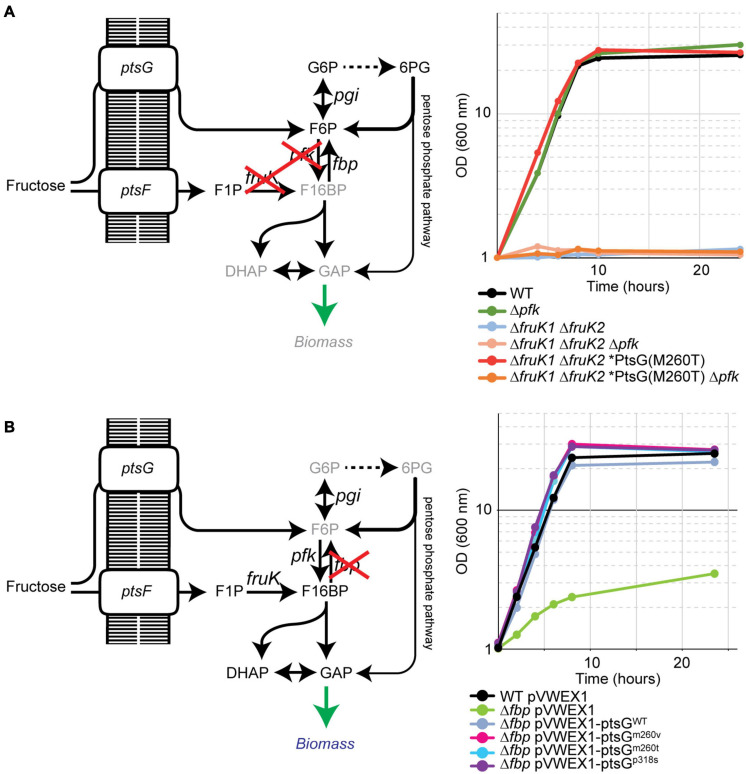
Genetic elucidation of fructose phosphorylation to fructose 6-P by PtsG. **(A)** Deletion of 6-phosphofruktokinase (Δ*pfk*) in Δ*fruK1* Δ*fruK2* fructose mutant abolishes growth. **(B)** Overexpression of PtsG variants improve growth of a fructose 1,6-bisphosphatase deletion strain (Δ*fbp*). Data represent one of two individual cultivations that differed <5%.

To test the hypothesis that PtsG phosphorylates fructose to yield fructose 6-P, genetic experiments were performed. First, it was determined if 6-phosphofructokinase is required for fructose catabolism *via* glucose-specific PTS. Therefore, the 6-phosphofructokinase gene (*pfkA*) was deleted in strain Δ*fruK1* Δ*fruK2*, which lacked both 1-phosphofructokinase genes, as well as in the evolved Δ*fruK1* Δ*fruK2* ALE mutant PtsG^*M*260*T*^. Both of these *pfkA* deletion mutants were not able to grow in fructose minimal medium (Figure 4A), indicating that PtsG phosphorylates fructose exclusively to fructose 6-phosphate.

The deletion of *pfkA* in the WT background did not alter growth of the strain with fructose, as fructose utilized *via* PtsF and FruK enters glycolysis at fructose 1,6-bisphosphate. In the absence of 6-phosphofructokinase in the strain Δ*fruK1* Δ*fruK2* and the Δ*fruK1* Δ*fruK2* ALE mutant PtsG^*M*260*T*^ (see Figure 4A), the only way fructose 6-P can be catabolized is *via* the oxPPP. From three molecules of fructose 6-P entering the oxPPP, one molecule of glyceraldehyde 3-phosphate (GAP) and three molecules of carbon dioxide are produced, while two molecules of fructose 6-P are regenerated. This low feed to the “lower” metabolism seemingly is not sufficient to allow for growth. One reason might be due to sugar phosphate stress. It might also be the case that metabolism probably does not utilize the GAP produced efficiently enough as it must be used to provide PEP for fructose phosphorylation, in order to keep the stoichiometric influx of fructose 6-P.

Based on previous findings that fructose 1,6-bisphosphatase is important for fructose catabolism *via* PtsF and 1-phosphofructokinases/FruK1 and/or FruK2 (Becker et al., 2005; Georgi et al., 2005), we hypothesized that strains growing on fructose *via* PtsG, synthesizing fructose 6-P directly from fructose, do not require fructose 1,6-bisphosphatase for growth. To test this, we overexpressed the PtsG variants in a Δ*fbp* strain and analyzed its growth on fructose (Figure 4B). This experiment tests only for the small flux to fructose 6-P, which is necessary to generate essential PPP intermediates (erythrose 4-P, ribose 5-P, and glucose 6-P). Notably, some residual growth on fructose was observed for Δ*fbp*. This might be, similar to Δ*ptsF*, due to the presence of the genomic *ptsG* in this strain, which is commensurate with some flux of fructose phosphorylation in the WT as observed previously (Kiefer et al., 2004). However, all PtsG variants allowed the Δ*fbp* strain to regain growth with fructose as fast as the WT strain (Figure 4B). Thus, fructose 1,6-bisphosphatase is dispensable for growth if fructose catabolism is mediated *via* PtsG with fructose being directly converted to fructose 6-P.

### Complementation of Δ*ptsG*Δ*ptsF* by *ptsG* Overexpression

**FIGURE 5:**
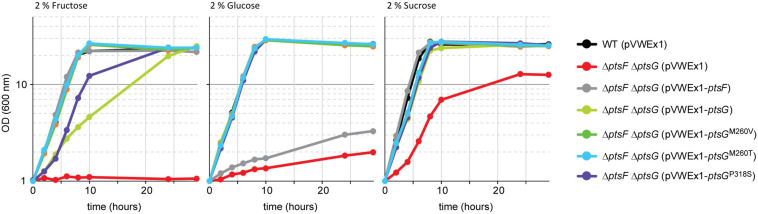
Growth complementation of the Δ*ptsF* Δ*ptsG* strain by overexpressing PtsG variants. Data represent one of two individual cultivations that differed <5%.

After having shown that PtsG-mediated fructose catabolism complemented the growth impairment due to the absence of fructose 1,6-bisphosphatase, a more rigorous test was attempted. A strain lacking the genes for both fructose- and glucose-specific PTS subunits was constructed (Δ*ptsG*Δ*ptsF*), and it was tested if PtsG and/or the selected PtsG variants support growth with fructose as the sole carbon source. Strain Δ*ptsF* Δ*ptsG* revealed a clean phenotype as growth with fructose as the sole carbon source was completely abolished (Figure 5). With glucose, however, Δ*ptsG*Δ*ptsF* showed some residual growth, which likely depended on PTS-independent glucose catabolism (Ikeda et al., 2011; Lindner et al., 2011). The observed reduction of OD to 50% with sucrose as the sole carbon source reflects the fact that only the glucose moiety can be utilized after its activation by PtsS and cleavage to glucose 6-P and fructose, but not the fructose moiety of the disaccharide, which can be catabolized by strain Δ*ptsF* Δ*ptsG*. Under the chosen conditions, PTS-independent fructose catabolism is irrelevant as indicated by this finding and the inability of strain Δ*ptsF* Δ*ptsG* to grow with fructose alone.

To test for *ptsG*-mediated fructose utilization, strain Δ*ptsG* Δ*ptsF* was transformed with expression plasmids overexpressing either native *ptsG*, one of the three newly identified *ptsG* variants, or, as positive control, native *ptsF*. Growth of the resulting strains in minimal media containing either fructose, glucose, or sucrose is shown in Figure 5. Growth of Δ*ptsF* Δ*ptsG* with glucose was complemented to a similar extent with all PtsG variants, including native PtsG, whereas overexpression of the fructose-specific PTS gene *ptsF* did not. On fructose, all constructs complemented the growth phenotype of Δ*ptsF* Δ*ptsG*, but to varying extent. Native *ptsG* supported a significantly lower growth rate (0.19 h^–1^) as compared to the growth achieved with the three mutated *ptsG*-versions (0.36–0.39 h^–1^) and with *ptsF* (0.44 h^–1^). Lag phases were observed only with native PtsG and with variant PtsG^*P*318*S*^ (Figure 5). Similar to the observed higher ODs reached by the ALE mutants as compared to their parent strains (Figure 3), the reverse-engineered strains analyzed also grew to about 20% higher OD in fructose minimal medium than strain Δ*ptsG*Δ*ptsF* (Table 3). With sucrose as the sole carbon source, all strains grew similar to WT except for Δ*ptsF* Δ*ptsG* carrying the empty vector as negative control, which showed a lag phase, grew slower, and reached about half of the OD compared to the other strains. Analysis of supernatants from sucrose grown Δ*ptsF* Δ*ptsG* revealed stoichiometric production of fructose from sucrose (data not shown).

**TABLE 3:** Growth rates and changes in OD (600 nm) during growth of recombinant strains on 2% glucose, 2% fructose, or 2% sucrose.

	**2% glucose**	**2% fructose**	**2% sucrose**
**Growth rates (h^–1^)**			
WT (pVWEx1)	0.40 ± 0.00	0.41 ± 0.01	0.49 ± 0.00
Δ*ptsF* Δ*ptsG* (pVWEx1)	0.03 ± 0.00	0.00 ± 0.00	0.26 ± 0.01
Δ*ptsF* Δ*ptsG* (pVWEx1-*ptsF*)	0.03 ± 0.00	0.45 ± 0.01	0.50 ± 0.01
Δ*ptsF* Δ*ptsG* (pVWEx1-*ptsG*)	0.37 ± 0.01	0.19 ± 0.01	0.33 ± 0.00
Δ*ptsF* Δ*ptsG* (pVWEx1-*ptsG*^*P318S*^)	0.40 ± 0.01	0.36 ± 0.00	0.41 ± 0.00
Δ*ptsF* Δ*ptsG* (pVWEx1-*ptsG*^*M260V*^)	0.40 ± 0.00	0.39 ± 0.01	0.42 ± 0.01
Δ*ptsF* Δ*ptsG* (pVWEx1-*ptsG*^*M260T*^)	0.40 ± 0.00	0.38 ± 0.00	0.42 ± 0.00
**ΔOD(600 nm)**			
WT (pVWEx1)	27.8 ± 0.2	21.1 ± 0.2	25.6 ± 0.2
Δ*ptsF* Δ*ptsG* (pVWEx1)	0.8 ± 0.2	0.0 ± 0.0	11.8 ± 0.0
Δ*ptsF* Δ*ptsG* (pVWEx1-*ptsF*)	25.6 ± 0.1	21.7 ± 0.5	25.8 ± 1.6
Δ*ptsF* Δ*ptsG* (pVWEx1-*ptsG*)	27.7 ± 0.5	25.1 ± 0.1	24.8 ± 0.6
Δ*ptsF* Δ*ptsG* (pVWEx1-*ptsG*^*P318S*^)	28.1 ± 0.3	24.6 ± 0.3	26.7 ± 0.3
Δ*ptsF* Δ*ptsG* (pVWEx1-*ptsG*^*M260V*^)	27.7 ± 0.7	24.8 ± 0.9	27.2 ± 0.4
Δ*ptsF* Δ*ptsG* (pVWEx1-*ptsG*^*M260T*^)	28.4 ± 0.0	25.6 ± 0.6	26.8 ± 0.4

### Faster Fructose Uptake Mediated by the Selected PtsG Variants

The three observed mutations M260T, M260V, and P318S are located within the EIIC permease subunit of the PTS-transporter domain, which mediates substrate translocation and transiently binds the substrate, until it becomes phosphorylated. In the well-characterized EIIC ChbC of *Bacillus cereus*, residues E334 and H250 interact with the substrate *N,N*′-diacetylchitobiose *via* hydrogen bonds (McCoy et al., 2015) and are located at the beginning of the transmembrane domain (TM) 7 and between TM8 and TM9, respectively. The topology of this region in ChbC is quite different from the (predicted) topology of BglF (beta-glucoside-specific) and MtlA (mannitol specific) EIIC proteins from *E. coli*. Based on the localization of predicted TM domains in *C. glutamicum* PtsG, the residues M260 and P318 are located as the first amino acid of TM5 and within TM6, respectively. P318 is very close to H322, which together with E389 are the two amino acids likely involved in substrate binding in *C. glutamicum* PtsG. Thus, the location of the mutations can be related to a change in substrate binding ability.

To analyze the kinetics of the PtsG-mediated fructose transport, we used ^14^C-labeled fructose as a tracer. The kinetic data obtained from these experiments are shown in Table 4. Strains that possess PtsF showed sigmoidal dependence of the uptake rate on the fructose concentration with Hill coefficients between 2 and 3, while Δ*ptsF* mutants did not (Table 4 and Supplementary Figure 3). No fructose uptake was detected by mutant Δ*ptsF* Δ*ptsG.* Fructose uptake was detected in the absence of PtsF; however, the *K*_*M*_ value was about 20-fold higher than the *K*_1__/__2_ value observed for strains that possess PtsF (Table 4). Moreover, fructose uptake was 5- to 10-fold faster in the presence of PtsF as compared to its absence. Thus, PtsF allowed for fast fructose uptake with high affinity, whereas PtsG supported slower uptake with lower affinity (Table 4).

**TABLE 4:** Kinetics parameters of fructose uptake by PtsF and PtsG.

**Strain**	**Hill coefficient**	**Sigmoid**	***K*_1__/__2_ (μM)**	***K*_*M*_ (μM)**	***V*_*max*_ {nmol/[min × mg (CDW)]}**
WT	3.00	Yes	45.7	–	73.5
Δ*ptsF*	/	X	–	841	8.4
Δ*ptsG*	2.38	Yes	44.8	–	41.6
Δ*ptsF* Δ*ptsG*	/	X	–	n.u.	–
Δ*ptsF* Δ*ptsG* (pVWEx1)	/	X	–	n.u.	–
Δ*ptsF* Δ*ptsG* (pVWEx1-*ptsF*)	2.57	Yes	30.0	–	40.8
Δ*ptsF* Δ*ptsG* (pVWEx1-*ptsG*)	/	X	–	739	6.7
Δ*ptsF* Δ*ptsG* (pVWEx1-*ptsG*^*M*260*V*^)	/	X	–	520	10.0
Δ*ptsF* Δ*ptsG* (pVWEx1-*ptsG*^*M*260*T*^)	/	X	–	459	7.1
Δ*ptsF* Δ*ptsG* (pVWEx1-*ptsG*^*P*318*S*^)	/	X	–	325	12.4

*n.u., no uptake.*

The PtsG variants showed higher affinity for fructose than WT PtsG. Graphs of fructose uptake are shown in Supplementary Figure 3. The lowest apparent *K*_*M*_ was determined for PtsG^P318*S*^ (325 μM), which is lower than half of WT PtsG (739 μM), but still 10-fold higher than the *K*_1__/__2_ value of PtsF. Two of the three PtsG variants (M260V and P318S) supported about 1.5- to 2-fold faster fructose uptake than WT PtsG {change of 6.7–10 and 12.4 nmol/[min × mg(CDW)], respectively}. Thus, the PtsG mutations showed improved kinetic parameters for fructose uptake as compared to WT PtsG. Notably, since the maximal uptake rates observed for the PtsG mutants did not reach that supported by PtsF, their improved kinetic parameters may not explain the fast growth observed for the respective strains *in vivo*. All growth experiments performed here exceed the *K*_*M*_ concentrations by more than 100-fold; thus, all PtsG variants should work under saturation conditions and affinity should not be a limiting factor.

### ^13^C-Labeling Experiments Reveal Substantially Higher oxPPP Flux

In order to analyze altered flux distributions in the strains utilizing fructose *via* PtsG, we performed ^13^C-labeling experiments. As the flux *via* the oxPPP and the associated NADPH provision is low during growth of *C. glutamicum* WT on fructose and depends at least in part on fructose 1,6-bisphosphatase, we hypothesized that PtsG-mediated fructose catabolism directly leading to fructose 6-P instead of fructose 1,6-BP might result in a higher flux *via* the oxPPP. To test this hypothesis, we performed ^13^C-labeling experiments with ^13^C-1-fructose as the sole carbon source for growth. For comparison, cells were grown with ^13^C-1-glucose. During fructose and glucose catabolism *via* the oxPPP, the labeled C1 is lost as ^13^CO_2_ in the oxidative decarboxylation of gluconate 6-P to ribulose 5-P. Thus, only unlabeled ribose 5-P is present and, hence, histidine, which is derived from ribose 5-P, is expected not to carry ^13^C label from the carbons derived from ribose 5-P. If the non-oxPPP is used to provide C_5_ building blocks, ^13^C-labeling of xylulose 5-P (generated by transketolase reactions) and other pentose phosphate molecules is expected. Specifically, the non-oxPPP converts two molecules of fructose 6-P (fully labeled at C1) and one molecule of GAP (50% labeling at C3) to two molecules of xylulose 5-P (fully labeled at C1 and 50% labeled at C3), and one molecule of unlabeled ribose 5-P as shown in detail in Supplementary Figure 4.

**FIGURE 6:**
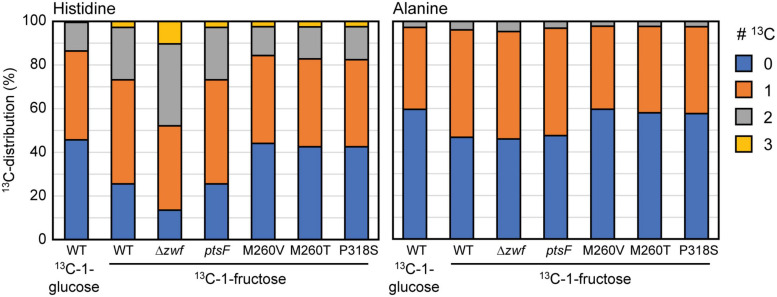
^13^C-labeling in L-histidine and L-alanine in WT, Δ*zwf*, and the Δ*ptsF* Δ*ptsG* strain overexpressing *ptsF* or the *ptsG* variants upon feeding ^13^C-1-fructose or ^13^C-1-glucose. Data represent means of three independent experiment errors <5%.

To obtain a clean negative control devoid of labeling patterns from the oxPPP, we included strain Δ*zwf*, which lacks glucose 6-P dehydrogenase, the entry point of the oxPPP. Figure 6 shows the labeling patterns of histidine and alanine in the ^13^C-labeling experiments performed with *C. glutamicum* WT and the indicated mutants. Our results confirmed previous findings that the relative flux *via* the oxPPP is lower during growth with fructose than during growth with glucose (Kiefer et al., 2004) since labeling in L-alanine and L-histidine was higher with ^13^C-1-fructose than with ^13^C-1-glucose. In the WT, the oxPPP is barely active during growth on fructose, indicated by the high ^13^C-labeling in L-alanine and L-histidine during growth with ^13^C-1-fructose, which was almost as high as in the Δ*zwf* strain, which lacks the oxPPP. The Δ*ptsF* Δ*ptsG* strain expressing *ptsF* showed a labeling pattern similar to WT, which indicated a low relative oxPPP flux when fructose was catabolized *via* PtsF. On the other hand, Δ*ptsF* Δ*ptsG* strains overexpressing the PtsG variants showed reduced absolute labeling of L-alanine and L-histidine. This provided evidence for a higher relative oxPPP flux when fructose is utilized *via* PtsG with direct conversion to fructose 6-P. Notably, the observed labeling is similar to the labeling observed when WT and the other strains grew on ^13^C-1-glucose (Figure 6 and data not shown). The absolute labeling in L-alanine and L-histidine (^13^C-abundance reduced by about 20% in Δ*ptsF* Δ*ptsG* strains overexpressing the PtsG variants compared to the Δ*zwf* strain used as reference lacking the oxPPP) allowed us to calculate that about 20% of the fructose molecules were catabolized *via* the oxPPP, which was comparable to the glucose utilized *via* the oxPPP in the WT.

### Evolved and Reverse-Engineered Strains Showed Reduced Overflow Metabolism

**FIGURE 7:**
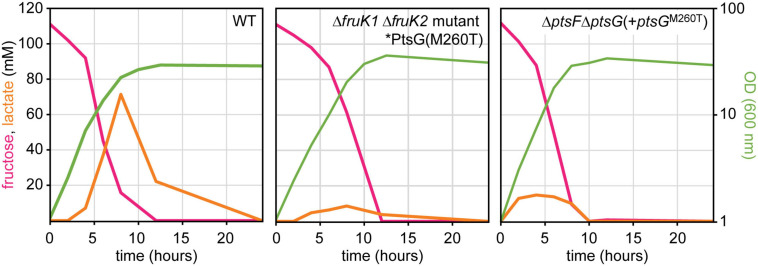
Transient lactate accumulation in cultures of WT, mutant, and reconstructed strain during growth on fructose. Data represent one of two individual cultivations that differed <5%.

Fast growth with glucose is known to be associated with intermittent lactate accumulation in the culture medium. During the exponential growth phase, NAD-dependent L-lactate dehydrogenase reduced pyruvate to L-lactate, which is secreted (Dominguez et al., 1998). L-lactate generation is significantly higher (fourfold) when fructose is the carbon source compared to glucose (Kiefer et al., 2004). L-lactate is re-utilized after induction of LldR by L-lactate and derepression of the *lld* operon for L-lactate catabolism (Georgi et al., 2008). The observed higher relative oxPPP flux when fructose is catabolized *via* the PtsG variants as compared to PtsF prompted us to investigate metabolic consequences. As already described above, the isolated mutants as well as the reverse-engineered strains grew to higher ODs than the *C. glutamicum* WT. Here, we investigated whether metabolic consequences can be observed with regard to by-product formation. Besides growing to higher ODs, the mutant and the reverse-engineered strain accumulated less L-lactate during growth (Figure 7).

### PtsG Catalyzed Fructose Utilization Improved L-Lysine Production

L-lysine production benefits from improved NADPH provision and reduced by-product formation. Since we have shown that PtsG-mediated fructose catabolism is characterized by increased oxPPP flux relevant for NADPH provision as well as reduced intermittent formation of L-lactate as by-product, the metabolic consequence on L-lysine production was determined. First, low-level L-lysine production by the ALE mutants was enabled *via* transformation using a plasmid for overexpression of a feedback-resistant aspartokinase gene (pVWEx1-*lysC*^*fbr*^). Compared to the control, i.e., *C. glutamicum* WT(pVWEx1-*lysC*^*fbr*^) L-lysine production in minimal medium containing 40 g/L fructose was increased about fivefold. The ALE mutants overexpressing *lysC*^*fbr*^ produced about 26 mM, while the WT overexpressing *lysC*^*fbr*^ produced 4.5 mM.

After these initial and very promising results, we constructed the PtsG pathway as the sole route for fructose utilization in the L-lysine producer CgLYS4 Δ*ptsF* (Sgobba et al., 2018). This strain is a *ptsF* deletion strain derived from CgLYS4 and, thus, lacks native fructose utilization *via* PtsF. CgLYS4 produces L-lysine due to feedback-resistant aspartokinase, attenuated homoserine dehydrogenase, and improved pyruvate carboxylase (*lysC^*T*311*I*^, hom^*V*59*A*^, pyc^*P*458*S*^*), and the strain shows reduced by-product formation as it carries deletions in the lactate dehydrogenase gene (*ldhA*) and acetate production genes (*pta*-*ackA*). Production of L-lysine was analyzed after growth with 40 g/L fructose as the sole carbon source and compared to the production achieved with the parental strain CgLYS4 (Sgobba et al., 2018). All tested strains utilizing fructose *via* PtsG accumulated about 50% more L-lysine than the PtsF-positive CgLYS4 (about 45 mM as compared to about 30 mM; Table 5). Notably, the strain overexpressing native PtsG reached a similar final L-lysine concentration to the strains overexpressing the new PtsG variants, but much later since this strain grew significantly slower (data not shown). Interestingly, overexpression of *ptsF* reduced L-lysine production compared to CgLYS4. Taken together, fructose catabolism *via* the isolated PtsG variants is a promising strategy to improve L-lysine production. These results might be of relevance for an increased bioproduction efficiency when using molasses as the feedstock, as molasses contain significant shares of fructose of their sugar compounds.

**TABLE 5:** L-lysine production of strains utilizing fructose *via* PtsG.

**Strain**	l-lysine (mM)
CgLYS4	29.7
CgLYS4 Δ*ptsF*^*a*^	0.8
CgLYS4 Δ*ptsF* pVWEx1-*ptsG*	43.1
CgLYS4 Δ*ptsF* pVWEx1-*ptsG^*P*318*S*^*	44.2
CgLYS4 Δ*ptsF* pVWEx1-*ptsG*^*M*260*V*^	46.9
CgLYS4 Δ*ptsF* pVWEx1-*ptsG*^*M*260*T*^	46.2
CgLYS4 Δ*ptsF* pVWEx1-*ptsF*	24.0

*Data represent means of three independent experiment with errors <5%. ^*a*^L-lysine produced by CgLYS4 Δ*ptsF* after 96 h of cultivation.*

## Discussion

In this study, ALE was used to isolate mutants able to catabolize fructose *via* PtsG. The PtsG variants enabled fast growth with fructose with increased relative oxPPP flux. Production of L-lysine was chosen as an application example and improved L-lysine titers associated with fast growth on fructose. However, it should be made clear at this point that fructose is not purely used in bioproduction of commodities and only makes a fraction of the sugar content of molasses (besides sucrose and glucose).

As shown here, only a few days of cultivation under selective conditions were sufficient to achieve the desired growth phenotype, indicating that mutation of a single gene was sufficient and that several mutations in this gene resulted in the desired growth phenotype. This is not unprecedented as earlier studies revealed the ability of *C. glutamicum* to evolve relatively quickly into a niche or to overcome a genetic impairment (Youn et al., 2009; Lindner et al., 2011; Uhde et al., 2013). The present study and the studies mentioned above share that they selected for utilization of a carbon and energy source. In addition, ALE has been used to select *C. glutamicum* mutants withstanding adverse conditions, e.g., due to methanol or indole (Lessmeier and Wendisch, 2015; Hennig et al., 2020; Kuepper et al., 2020; Walter et al., 2020) or mutants that have overcome the requirement for an additive such as iron chelator PCA (Graf et al., 2019) or production of, e.g., putrescine (Jorge et al., 2017; Li and Liu, 2017). Thus, ALE has proven valuable for *C. glutamicum* metabolic engineering (Stella et al., 2019).

Apart from *C. glutamicum*’s PtsG, the mannose PTS system of *E. coli* also generates fructose 6-P from fructose (Kornberg, 2001); this tendency of promiscuity of the PTS system compounds also applies to some sugar kinases, e.g., *E. coli*’s enzymes xylulokinase is active with xylulose and ribulose (Di Luccio et al., 2007) and fuculokinase is active with fuculose and ribulose (LeBlanc and Mortlock, 1971). One explanation for their promiscuity is that carbohydrate kinases are ancient enzymes, which needed to evolve into niches of present carbon sources (Roy et al., 2019). A good example for enzyme promiscuity is the fast evolution for utilization of new substrates shown for *E. coli* (Guzman et al., 2019). Regarding PTS specificity, a prominent and promiscuous example is probably *E. coli*’s mannose PTS compound, which, besides mannose, also takes glucose, fructose, N-acetylglucosamine, and glucosamine (Curtis and Epstein, 1975; Chou et al., 1994). Similar to the approach described here, Wang et al. (2016) used a *C. glutamicum* Δ*ptsF* strain, evolved it on sucrose, and found suppressor mutants with inactivated 1-phosphofructokinase gene, indicating the role of sugar phosphates in transcriptional repression, likely of *ptsG*, which might explain the enhanced NADPH and L-lysine production from sucrose and fructose. The responsible regulator of sugar utilization, SugR, represses expression of *ptsG* (Engels and Wendisch, 2007), and deletion of *sugR* derepressed *ptsG* transcription and consequently facilitates glucose utilization and improved L-lysine productivity (Perez-Garcia et al., 2016).

The results deduced from the ^13^C-labeling obtained in our study show some differences to the data described by Kiefer et al. (2004), which might be explained by the use of an L-lysine producer strain in the study by Kiefer et al. (2004) since L-lysine overproduction provides a strong NADPH sink. *C. glutamicum* can respond to different metabolic burdens differing in their NADPH requirements, as was shown in a metabolic flux comparison of *C. glutamicum* WT grown either under standard conditions or upon triggering L-glutamate production and of an L-lysine-producing strain (Marx et al., 1997). Flux in the oxPPP and, thus, NADPH generation was highest in the L-lysine producer, intermediate in WT and lowest under L-glutamate production (Icd provides NADPH and 2-oxoglutarate to balance the NADPH requirement of glutamate dehydrogenase for reductive amination of 2-oxoglutarate to yield glutamate) (Bormann et al., 1992).

We used L-lysine production as a readout to prove the increased NADPH availability in the engineered strains. It is important to state that all experiments were carried out in shake flasks and a transfer to a robust bioreactor culture is needed as the first step of up-scaling experiments to describe oxygen transfer, pressure, and foaming as highly relevant parameters that do not scale well when transferring from shake flasks to technical scale bioreactors (Takors, 2012). In addition to its NADPH demand, L-lysine production highly depends on strong fluxes toward anaplerosis, providing oxaloacetate as the precursor for aspartate biosynthesis, the starting point of L-lysine biosynthesis. The supply of anaplerotic precursors might be negatively affected by the PEP-dependent sugar phosphorylation carried out by the PTS system, as PTS-independent sugar utilizations improved L-lysine production (Lindner et al., 2011). However, using ATP-dependent sugar phosphorylation, e.g., fructokinase was shown to have a negative effect on ATP availability and hence sugar uptake (Xu et al., 2020). Recently, for L-lysine and L-threonine production (both high-NADPH demanding products), optimal flux ratio between oxPPP and glycolysis was determined (Murai et al., 2020), indicating a high demand of oxPPP for these products. Similar to the effects seen for L-lysine, our discovery might be of value for biotechnological use for high-NADPH-dependent products, e.g., threonine or 1,5-diaminopentane. This approach may be paired with others: Further approaches tackling NADPH recovery for increased bioproductions are overexpression of membrane-bound transhydrogenase (Kabus et al., 2007), deletion of phosphoglucose isomerase (Marx et al., 2003), overexpression of NAD kinase (Lindner et al., 2010), and overexpression of oxPPP enzymes (Becker et al., 2007). However, these studies exclusively focused on glucose as a carbon source. Indirect effects similarly to the approach described above were undertaken by overexpressing gluconeogenetic fructose 1,6-bisphosphatase, which increased L-lysine production from fructose (Georgi et al., 2005).

A new and fast way of fructose utilization *via* the optimized PtsG variants was shown in the biotechnologically important microbe *C. glutamicum*. The pathway supports higher relative oxPPP flux and consequently an improved NADPH regeneration rate, which was exploited here for the high NADPH demanding L-lysine production. The here described results and especially the PtsG mutations might also be advantageous for the production of other NADPH demanding products, e.g., other amino acids or derivatives like diamines, and fatty acids.

## Data Availability Statement

The raw data supporting the conclusions of this article will be made available by the authors, without undue reservation.

## Author Contributions

SNL and VFW conceived the study, designed the experiments, and analyzed the results. IK, DB, LT-B, JPK, TMM, and SNL performed metabolic engineering experiments. NR and GMS performed sugar uptake experiments. SNL, VFW, and GMS wrote the manuscript with contributions from all authors. All authors agreed with the final version of the manuscript.

## Conflict of Interest

The authors declare that the research was conducted in the absence of any commercial or financial relationships that could be construed as a potential conflict of interest.
